# Influence of Water Glass Introduction Methods on Selected Properties of Portland Cement

**DOI:** 10.3390/ma14123257

**Published:** 2021-06-12

**Authors:** Wiktor Szewczenko, Galyna Kotsay

**Affiliations:** Faculty of Civil Engineering, Mechanics and Petrochemistry, Warsaw University of Technology, 17 Łukasiewicza St., 09-400 Płock, Poland; galyna.kotsay@pw.edu.pl

**Keywords:** cement, admixture, water glass, setting time, impact strength, compressive strength

## Abstract

This article presents a study of the effect of water glass and its introduction on the hydration of Portland cement and its properties in plastic and solid states. The introduction of sodium water glass into the mixing water extends the setting time of Portland cement by 35%, while introduction into the cement paste reduces it by 24.4%; for potassium water glass, the respective values are 10.8% and 10.8%. The introduction of sodium water glass into the mixing water decreases its consistency by 17.6%; its introduction into the cement paste reduces its consistency by 97%. Based on microcalorimetric studies and using the modelling method, mechanisms of the processes occurring in the cement paste, for various methods of introducing water glass admixtures, and their influence on the properties of cement are proposed. The important implications of the obtained results are that, using various methods for introducing admixtures of water glass, it is possible to regulate the setting of cement slurries within significant limits that are important during their transportation.

## 1. Introduction

In the construction industry, admixtures that accelerate setting and hardening are used to produce reinforced concrete products and sprayed concrete, and to compensate for low temperatures during concreting in the winter [1,2,3,4]. Inorganic accelerating admixtures include alkaline ones—hydroxides, carbonates, aluminates, silicates, nitrates, sulfates, and sodium and potassium thiosulfates—and non-alkaline ones: aluminum sulfate or aqueous solutions of complex aluminosulfate salts [2,5,6,7,8,9]. Each of these admixtures has its advantages and disadvantages; for example, carbonate impurities are harmful for electroplating in concrete [10], but harmless to steel. It is known that admixtures that accelerate the hardening of cement can reduce the final strength of cement products [11,12]. For example, alkaline accelerators hasten the setting of cement paste but reduce the long-term strength by 50–60% [13]. Additionally, the results of using accelerators are highly dependent on correct and accurate dosing. Inappropriate dosing can lead to the corrosion of steel reinforcements, a decrease in the strength of the concrete, and, in some cases, the reverse of what is intended: inhibition of the hardening process. One way to solve this problem is to use fast-setting cement.

Unlike accelerators, retarding admixtures slows down the hydration of cement and increases the setting and hardening times. According to standard EN 934-2 [14], an admixture for increasing the setting time should extend the initial setting time by at least 90 min. In this case, the final setting time should be no more than 360 min longer than that for a control sample, and the compressive strength of concrete (for example) with such an admixture should be at least 80% after 7 days of hardening and 90% after 28 days of the control sample’s compressive strength. Such admixtures essentially work by slowing down cement hydration. The mechanisms by which the admixtures can inhibit cement hydration are various—e.g., the formation of difficult-to-dissolve compounds, the adsorption of large organic molecules, or the formation of silica gel on the surface of cement grains [15,16]. Such admixtures simultaneously decrease the mechanical strength in the early stages of hardening, but that increases with time.

The analysis of admixtures that accelerate and delay the start of the setting and hardening processes for cement products reveals the following common disadvantages: the use of chemical compounds, most of which have toxic properties; the instability of the action of several admixtures with different purposes introduced simultaneously; the need for accurate dosing regardless of the conditions for preparing the concrete mixture; a possible reduction in mechanical strength in the early stages of the hardening of cement products.

In the last 20 years, relatively many studies have appeared on the use of glass waste as additives for Portland cement [17,18,19,20,21,22,23,24]. Container glass of various chemical compositions is most often used: colorless, green, and brown. These glasses differ in chemical composition but all contain 13–14% Na_2_Oeq [25]. The alkaline activity of such additives is high and depends on the size of the glass grains. The most active is the fraction <0.063 mm with Blaine surface ≥ 3000 cm^2^/g [26]. The authors of [25] found that mixing water extracts the alkali from the glass and causes an increase in the alkalinity of the cement paste.

It is indicated in [27] that glass particles can act as nucleating agents at the initial stage of the crystallization of a cement stone by a heterogeneous mechanism. The possibility of using water glass as an admixture with a molecular dispersion is shown, i.e., as a nano-additive. In construction, water glass with a siliceous modulus of 2.7–2.9 is used for acid-resistant fillers and mortars, protective coatings, and chemical admixtures for concrete to reduce its setting time [28,29,30,31,32]. However, the functional properties of the admixture are not unambiguous. To understand the consequences of the interaction of the admixture with the cement, it is necessary to understand the principle of its action. Water glasses are aqueous mixtures of sodium or potassium silicates and their hydrolysis products [29,32,33]. Depending on the alkalinity of the solution, silicate anions in water glass have different degrees of polycondensation. When cement is mixed with water, the liquid phase of the paste becomes saturated with calcium ions, and the pH of the cement paste rises to 12–13. Consequently, the introduction of water glass, which has a high alkaline activity, should help to increase the pH of the solution and accelerate the cement hydration. However, by changing the method of introducing water glass, it is possible to obtain a SiO_2-_ gel, which, on the contrary, slows down the hydration process. To clarify these contradictions, studies were carried out to determine the effect of the method of introducing liquid glass on the hydration and hardening of Portland cement and its properties.

## 2. Materials and Methods

This study used sodium and potassium water glasses (WG) produced by the Chemical Plant “Rudniki” S.A (Rudniki, PL). [34]; their characteristics are presented in Table 1.

Portland cement type CEM I 42.5N, produced by Group Cement Ożarów (Ożarów, Poland) [35], was used as the main object of research. The composition and properties of cement are presented in Table 2. All the samples were prepared in distilled water. A Calmetrix 1-Cal 2000 HPC microcalorimeter (Calmetrix Inc., Boston, MA, USA) was used to study the process of cement hydration. For all the investigated compositions, a water–cement ratio (W/C) of 0.5 was used because only such a ratio made it possible to qualitatively mix water with cement quickly, which was necessary for analysis.

The initial and final setting times for the cement pastes were determined automatically with the Vicatronic apparatus from Matest (Ożarów Mazowiecki, Poland). The consistency of the cement paste was determined according to the European standard EN 196-3 [36] and was assessed by the depth of immersion of a metal rod with a diameter of 10 mm and a mass of 300 g.

The mechanical strength was determined on specimens—beams that were 40 × 40 × 160 mm^3^ in size—in accordance with the standard [37]. The impact strength was determined in accordance with the standard [38]. Compressive strength tests were performed on six specimens. The impact strength tests were performed on 28 specimens. The strengths of the samples were determined at 1, 2, and 28 days of curing.

## 3. Experimental Section

Water glass was introduced into the cement paste and mortar composition in amounts of 2, 5, and 8 wt % relative to the weight of the cement. Table 3 shows various options for introducing sodium and potassium water glasses into cement pastes: water glass previously introduced into mixing water (options N2 and N4) and into a water–cement mixture after 15 s of mixing cement with water (options N3 and N5).

**Figure 1:**
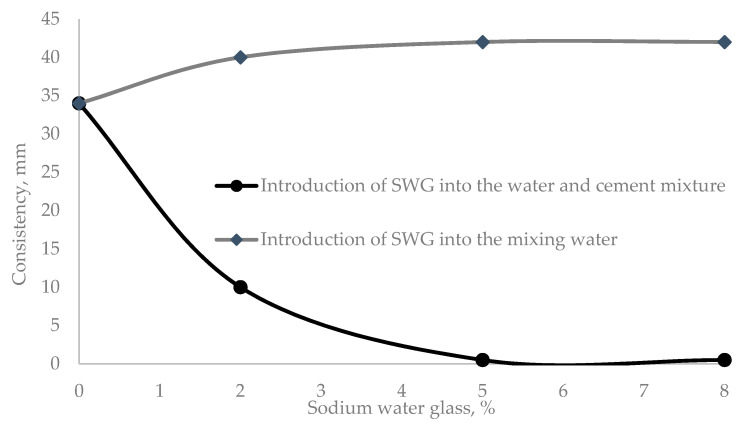
Dependence of the consistency of the cement paste on the amount of WG admixture and the method of its introduction.

The difference in the method of introducing water glass in the preparation of cement paste and mortar is because, in the first variant, WG participates in the hydration in the pre-induction period. In the second variant, WG is introduced into the composition of the water–cement mixture at a later stage of hydration, in the induction period. Such a difference in the method of WG administration is almost directly reflected in the consistency of the water–cement mixture, which increases when water glass is added to the water–cement mix (Figure 1).

The most significant changes in the consistency of the cement paste in comparison with the control sample (35 mm) were observed when 2% sodium water glass was added (Figure 1). When the admixture was added to the mixing water, the consistency decreased by 15%, while when it was added to the water–cement mixture, the consistency increased by 28.6%, as reflected in the cement setting parameters (Table 4).

The results presented in Table 4 show that the introduction of 5% sodium WG into the mixing water increased the time to initial setting by 130 min, while its introduction into the water–cement mixture reduced it by 90 min. For potassium water glass, the introduction of 5% WG into the mixing water increased the initial setting time by 40 min. Its introduction into the water–cement mixture reduced it by 40 min.

**Figure 2:**
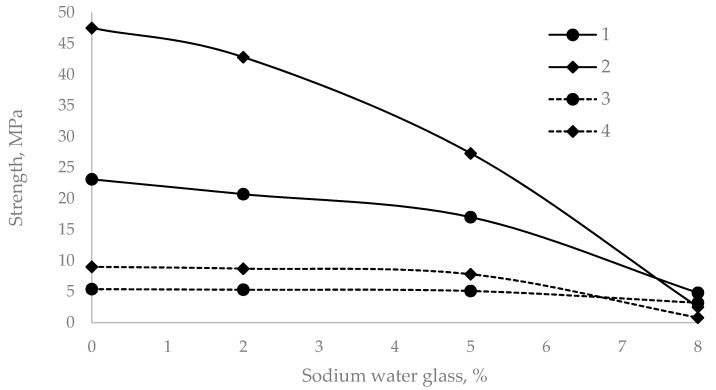
Compressive and flexural strength of cement mortars with different sodium water glass dosages. (1 and 2—compressive strength after 2 and 28 days; 3 and 4—flexural strength after 2 and 28 days.).

The admixture of water glass and an increase in its amount led to a decrease in the mechanical compressive and tensile strength compared with those of the control sample (Figure 2).

As shown in Figure 2, when 2% sodium water glass was added, the compressive strength was reduced by 10% and the flexural strength was reduced by 2% after two days of curing. After 28 days, these values were 1.7% and 3.4%, respectively. With 5% water glass, the reduction in compressive strength after two days was 26.4%, and that in tensile strength was 5.6%. After 28 days, these values were 18.1 and 13.4%, respectively. A 2% amount of additive can be considered optimal, considering that the standard allows a 10% reduction in strength.

**Figure 3:**
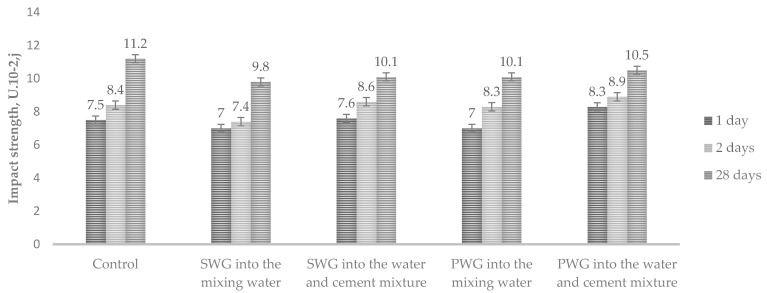
Impact strength depending on the method of WG introduction.

To determine the effect of introducing water glass admixtures on Portland cement’s mechanical properties, the impact strength for cement paste and compressive and flexural strength in bending for cement mortar were studied. A 6% decrease in impact strength was observed when sodium and potassium water glasses were introduced into the mixing water. Adding the admixtures of water glass into the water–cement mixture increased the impact strength by 3–6% (Figure 3).

**Figure 4:**
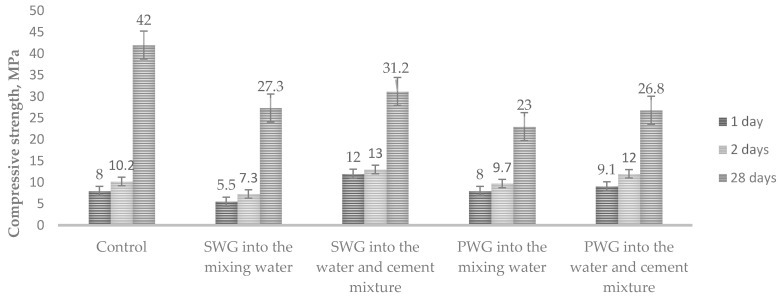
Influence of water glass on the compressive strength cement of mortars with water glasses.

The compressive strength was determined at the same time (Figure 4).

The results show that the compressive strength of the cement mortars was two times lower when the admixtures of sodium water glass were introduced into the mixing water than when they were introduced into the water–cement mixture. For potassium water glass, the difference was slightly lower. After 28 days, the strength of all the samples was significantly lower than that of the control samples.

As already discussed, an increase in the strength of cement over time is associated with its hydration, which is an exothermic process. The amount of heat released depends on the type of cement and the additive impurities it contains. In the early stages of hydration, there are prerequisites for obtaining specific properties in Portland cement. Therefore, microcalorimetric analysis is the most suitable method for analyzing the processes of cement hydration at its early stages.

**Figure 5:**
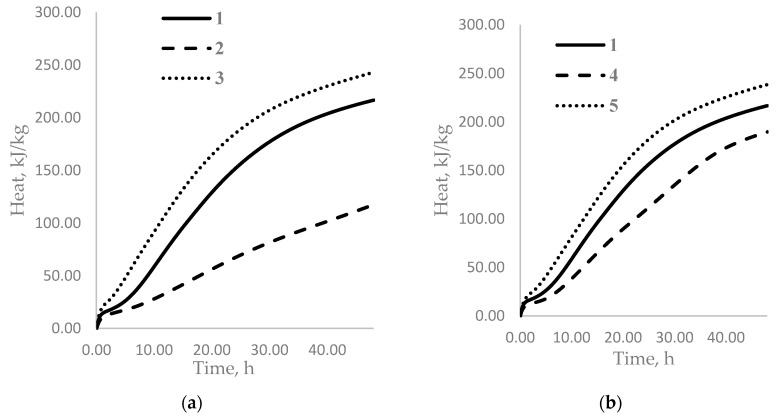
Heat curves for mixtures of cement with 5% sodium and potassium water glasses. 1—100% CEM; 2—100% CEM + 5% SWG into the mixing water; 3—100% CEM + 5% SWG into the water and cement mixture; 4—100% CEM + 5% PWG into the mixing water; 5—100% CEM + 5% PWG into the water and cement mixture. (**a**) CEM + sodium WG; (**b**) CEM + potassium WG.

Figure 5 shows the dependence of the amount of heat released on the time of cement hydration with the addition of WG at different stages of the preparation of the cement paste. Microcalorimetric analysis was carried out over 48 h, which corresponds to the early period of hydration.

As shown in Figure 5a, regardless of the method of introducing water glass after one hour of hydration, the amount of heat generated was approximately the same for the composition with water glass and the control sample. After 10 h, the amount of heat released under the condition of introducing sodium water glass into the mixing water decreased by 44% compared to that for the control sample and increased by 56% when the water glass was added to the water–cement mixture. After 48 h, these values were 53% and 11%, respectively. A similar picture was observed with the potassium water glass (Figure 5b). The only difference is that, after 10 and 48 h, the heat release was higher, compared to that for the control sample, than that with the sodium water glass.

A comparison of the maximum rates of heat release in all the periods of hydration shows that the addition of water glass impurities to the mixing water reduced the rate of heat release at the post-induction stage of hydration by almost two times in comparison with the introduction of water glass into the cement paste (Table 5).

**Figure 6:**
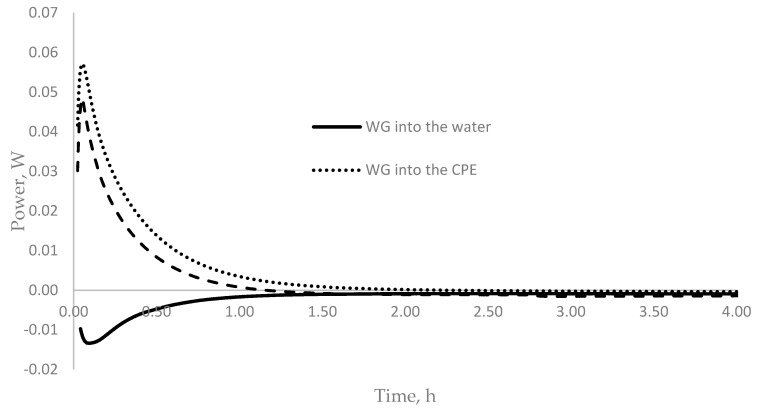
Time dependence of heat release when sodium WG was exposed to various reagents included in cement hydration products.

Based on the data presented, it can be assumed that the effect of water glass on the hydration of cement depends on its method of introduction. To understand the reason for this phenomenon, it is necessary to simulate exposure to WG in the environment during cement hydration. The construction of the corresponding model was based on the microcalorimetric analysis of the action of sodium WG, separately, on each of the reagents formed during the hydration of Portland cement and their joint presence in the composition of the obtained cement paste extract (CPE), according to the method described in [39]. Figure 6 shows the dependence of heat release on the time for each reagent separately and during their combined action.

When water glass was introduced into the mixing water, a sharp decrease in the rate of heat release was observed (Figure 6), which may indicate the dissolution of water glass with the formation of hydrolysis products according to the reaction

Na_2_O·nSiO_2_·mH_2_O + H_2_O → 2NaOH + H_2_SiO_3_ (1)

The dependence of the amount of heat released on the method of introducing water glass is associated with the characteristic of SiO_2_ coagulation in various aqueous media [33,40,41]. In a neutral medium (mixing water), SiO_2_ coagulation occurs at a maximum rate, while an alkaline medium helps to stabilize the SiO_2_ zol in solutions. Figure 6 shows that, when sodium WG was added to water, heat absorption was observed, indicating the coagulation of the SiO_2_ gel due to Reaction (1). Therefore, when water glass was introduced into mixing water (which has a pH of 7), the WG released a SiO_2_- gel, which enveloped the cement grains and impeded water access. This led to a decrease in the rate of hydration and delayed the onset of cement setting (Table 4).

A completely different picture emerges under the action of a saturated solution of calcium hydroxide and CPE on sodium WG (Figure 6). Within 0.1 h, a slight exothermic effect was observed, which may be associated with the formation of intermediate products, and this decreased to zero over the next two hours. When WG was added to the cement paste, which has a high pH value, SiO_2_ coagulation became difficult, which led to the reaction of WG with calcium hydroxide, with the formation of alkali calcium-silicate-hydrate (N(K)CSH) product gel, which was accompanied by an intense release of heat (Figure 6).

The formation of an alkaline gel upon introducing water glass into the cement paste contributes to a decrease in cement samples’ mechanical strength compared with control samples [11]. The research results presented in the article make it possible to assert that the addition of water glass, regardless of its type, significantly affects the hydration and hardening of Portland cement. When water glass is introduced into mixing water, the hydration process slows down due to the formation of a SiO_2_- gel, which leads to a decrease in mechanical strength compared to the control samples. When introduced into a water–cement mixture, water glass reacts with calcium hydroxide to form an alkaline gel, contributing to a decrease in mechanical strength compared with control samples, but a higher strength than when WG is added to the mixing water.

When using water glass admixtures, it is necessary to consider the increase in the alkaline activity of the cement paste. It is known that Portland cement has a high potassium alkaline activity. Therefore, considering the mixed alkaline effect, which involves the introduction of another type of alkaline component, it is desirable to use sodium water glass to suppress the alkaline potassium activity of Portland cement [42].

## 4. Conclusions

This study shows the possibility of controlling the setting time of Portland cement using water glass as an admixture. Having a high alkaline activity, water glass has a significant effect on the hydration and hardening of Portland cement. It has been established that the introduction of water glass into the mixing water extends the setting time of Portland cement by 120 min, and introduction into the cement paste, on the contrary, reduces it by 90 min; however, for the potassium water glass, these times are 40 min. Studies of the mechanical strength of samples with water glass showed that the impact strength and compressive strength decreased with an increase in the amount of added water glass. For example, after two days of hydration, with an increase in the amount of water glass from 2% to 8%, the compressive strength decreased by 4.3 times, and after 28 days, 17 times. As shown by microcalorimetric studies using the modelling method, when water glass, regardless of its type, is introduced into the mixing water, a SiO_2_ gel is formed, which inhibits the hydration process. An N(K)CSH gel is created, which loosens the cement structure, reducing its mechanical strength. When water glass is introduced into the cement paste, the calcium hydroxide formed during cement hydration interacts with the sodium (potassium) metasilicate of the water glass to form an additional N(K)CSH gel, increases the consistency of the cement paste, and shortens the setting time.

Considering that it is possible to regulate the setting time of Portland cement while insignificantly affecting the strength of the cement products (within the limits allowed by the standards), in a manner that depends on the method of introducing the water glass, 2% can be considered an optimal amount of water glass to add.

## Figures and Tables

**Table 1:** Characteristics of sodium and potassium water glasses.

WG	Chemical Composition of Water Glass, wt %			Siliceous Module
	SiO_2_	Na_2_O	K_2_O	
Sodium	26.14	7.86	-	3.32
Potassium	23.56	-	6.58	3.58

**Table 2:** Composition and specific surface of Portland cement.

Chemical Composition, wt %		Phase Composition ^1^, wt %		Blaine Surface (cm^2^/g)
CaO	64.23	Alite (s)	60.40	3380
SiO_2_	21.75	Belite (b)	17.42	
Al_2_O_3_	3.54	Tricalcium aluminate	3.47	
Fe_2_O_3_	3.50	Tetracalcium aluminoferrite	10.60	
Na_2_O_eq_	0.71	Gypsum	5.25	
MgO	0.80			
SO_3_	2.44			
Los of ignition	3.03			

^1^ Composition phase (Bogue).

**Table 3:** Various options for introducing sodium and potassium water glasses.

N Position	Composition	Introduction of WG
1	100% CEM	-
2	100% CEM + 5% sodium water glass (SWG)	Into the mixing water
3		Into the water and cement mixture
4	100% CEM + 5% potassium water glass (PWG)	Into the mixing water
5		Into the water and cement mixture

**Table 4:** Results of the setting times of cement pastes, depending on the method of introducing water glass.

N Position	Setting Time, min	
	Initial	Final
1	370	410
2	500	680
3	280	320
4	410	490
5	330	380

**Table 5:** Rates of heat release in all periods of hydration depending on the method of WG introduction.

N	Maximum Rate of Heat Evolution, W/kg		
	Pre-Induction Stage of Hydration	Induction Stage of Hydration	Post-Induction Stage of Hydration
1	8.18	0.62	2.16
2	4.54	0.41	0.81
3	10.45	1.36	2.51
4	5.35	0.43	1.25
5	9.24	1.14	2.28

## Data Availability

The data presented in this study are available on request from the corresponding author.
